# Ameba-associated Microorganisms and Diagnosis of Nosocomial Pneumonia

**DOI:** 10.3201/eid1202.050434

**Published:** 2006-02

**Authors:** Pierre Berger, Laurent Papazian, Michel Drancourt, Bernard La Scola, Jean-Pierre Auffray, Didier Raoult

**Affiliations:** * Centre Hospitalier Universitaire La Timone, Marseille, France;; † Université de la Méditerranée, Marseille, France;; ‡ Hôpital Sainte-Marguerite, Marseille, France

**Keywords:** Pneumonia, ameba-associated microorganisms, laboratory techniques and procedures, intensive care unit, prospective studies, research

## Abstract

To elucidate the role of ameba-associated microorganisms (AAMs) as etiologic agents of pneumonia, we screened for *Legionella* spp., *Parachlamydia acanthamoeba*, *Afipia* sp., *Bosea* spp., *Bradyrhizobium* spp., *Mesorhizobium amorphae*, *Rasbo bacterium*, *Azorhizobium caulinodans*, *Acanthamoeba polyphaga mimivirus*, and conventional microorganisms in 210 pneumonia patients in intensive-care units by using culture, polymerase chain reaction, and serologic testing. These resulted in 59 diagnoses in 40 patients. AAMs and non-AAMs were implicated in 10.5% of the patients. The infectious agents were identified in 15 patients: *Acanthamoeba polyphaga mimivirus*, 8; *Legionella pneumophila*, 3; *L*. *anisa*, 1; *Parachlamydia* sp., 1; *Bosea massiliensis*, *L*. *worsleiensis*, *L*. *quinlivanii*, and *L*. *rubrilucens*, 1; and *M*. *amorphae* and *R*. *bacterium*, 1. *A*. *polyphaga mimivirus* was the fourth most common etiologic agent, with a higher seroprevalence than noted in healthy controls. This finding suggested its clinical relevance. Therefore, AAM might cause nosocomial pneumonia and should be suspected when conventional microbiologic results are negative.

## Abstract 2

Ameba-associated microorganisms should be suspected when conventional microbiologic test results are negative.

Pneumonia is a major cause of illness and death throughout the world (*1*). Approximately 600,000 persons with pneumonia are hospitalized each year, and 64 million days of restricted activity occur because of this disease (*2*). Pneumonia is associated with high death rates, in particular, 30% for community-acquired pneumonia (*3*). Hospital-acquired pneumonia occurs in 0.5% to 1% of hospitalized patients, which represents 10%–15% of all nosocomial infections; pneumonia is the most common cause of nosocomial infection in intensive-care units (ICUs) (*4*). The etiologic agent of community-acquired pneumonia remains unknown in 20% to 50% of cases (*5*), and several pathogens that may cause pneumonia seem to be underestimated (*6**–**8*).

Microbiologically contaminated water distribution systems have been linked to outbreaks of hospital- and community-acquired pneumonia (*9**,**10*). Water-associated microorganisms, such as *Legionella* spp., *Pseudomonas* spp., *Stenotrophomonas* spp., *Burkholderia* spp., and *Acinetobacter* spp., colonize hospital water supplies and have been causally associated with cases of hospital-acquired pneumonia (*10*). There is also a growing concern that water–associated microorganisms, for example, *Legionella* spp., *Afipia* spp., *Bosea* spp., *Bradyrhizobium* spp., *Mesorhizobium* spp., *Rasbo bacterium*, *Parachlamydia* spp., and *Acanthamoeba polyphaga mimivirus*, may be associated with amebas (*11**–**13*). We previously demonstrated that patients with nosocomial pneumonia who received care in a hospital near a contaminated water distribution system showed strong serologic evidence of exposure to these microorganisms (*14*). Specimens from 12 (40%) of 30 patients in an ICU seroconverted to microorganisms known to survive in an aquatic environment in the intracellular niche provided by free-living *Acanthamoebae* (*15*). These seroconversions were associated with ventilator-associated pneumonia, especially in patients for whom no etiologic agent was found by usual microbiologic investigations. We have also reported serologic evidence of exposure to an emerging giant virus that is resistant to phagocytic destruction by ameba, which we named *A*. *polyphaga mimivirus* (http://www-micro.msb.le.ac.uk/3035/VirusGroups.html), in 26 patients with ventilator-associated pneumonia at another ICU (*12**,**13**,**16*). Using this rationale, we evaluated infections with ameba-associated microorganisms (AAM) in a larger series of patients with pneumonia hospitalized in Marseille, France. Our goal was to identify typical pathogens as well as emerging AAMs (*12**–**15**,**17**–**20*).

## Materials and Methods

### Study Population

All patients admitted to the ICU of Sainte-Marguerite Hospital in Marseille, France, with clinically suspected pneumonia over an 18-month period ending in June 2003 were enrolled in a prospective study. For all patients, the clinical suspicion of pneumonia was based on the presence of new or progressive pulmonary infiltrates on chest radiograph along with 2 of the following features: pyrexia with a temperature >38.5°C, purulent tracheobronchial secretions, and leukocytosis with a total peripheral leukocyte count >12,000/mm^3^. All episodes of suspected ventilator-associated pneumonia with fever and pulmonary density were retrospectively reevaluated and all differential diagnoses were excluded (*21*). Since patients might have been treated for pneumonia more than once during the 18-month study period, episodes rather than individual patients were the unit of analysis. Beginning from the time of admission, all occasions on which pneumonia had been diagnosed in individual patients were considered a single episode of pneumonia unless the interval between 2 such occasions exceeded 30 days. Excluded from the final analyses were patients who did not recover between 2 episodes of pneumonia.

### Data Collection

Samples used for this study resulted from the current residual sampling strategy of the ICU; no supplement sampling was performed for the study. The identity of patients who provided samples and questionnaire information before the study remained confidential according to French law. We collected clinical data by using a standardized questionnaire that included sociodemographic data (age, sex), medical history (chronic obstructive bronchopneumonia, asthma, cystic fibrosis, smoking and alcohol habits, immunosuppression, cancer, HIV infection, malnutrition, tuberculosis), hospitalization data (surgery, inhalation therapy, duration of ventilation, and antimicrobial drug use), and type of pneumonia (nosocomial or community acquired). Acute respiratory distress syndrome (ARDS) was defined according to the American-European consensus (*22*). Information on immunosuppression was obtained for patients with a history of cancer, organ transplants, splenectomy, HIV infection (when the CD4+ T-cell count was <200 cells/μL), and immunosuppressor or steroid treatment (>0.5 mg/kg prednisone for >30 days or >5 mg/kg prednisone for >5 days).

### Study Design

The diagnostic strategy included bronchoalveolar lavage (BAL) fluid, blood cultures, and serologic and urine samples. BAL was performed by wedging the bronchoscope into a subsegment of the area of the lung when greatest abnormality was seen on a radiograph, or when the disease was diffuse, into the lingual or right middle lobe. Normal saline was sequentially instilled in 20-mL aliquots and sectioned into sterile traps for microbiologic testing for AAMs. This testing included culturing onto an agar base containing buffered charcoal yeast extract and enriched with α-ketoglutarate and L-cysteine (*23*) (Oxoid, Dardilly, France) with cefamandole, polymyxin B, and anisomycin for *Legionella* spp. cultivation; coculture with amebas as previously reported (*24**,**25*) for AAM; and TaqMan real-time polymerase chain reaction assay for enhanced detection of AAMs (*Legionella pneumophila*, *L*. *anisa*, *Parachlamydia* spp., *Bosea* spp., and *A*. *polyphaga mimivirus*). DNA was extracted from BAL samples by using the QIAMP tissue kit (Qiagen, Hilden, Germany) according to the manufacturer's instructions. Acute- and convalescent-phase serum samples were drawn into vacutainer tubes (Becton Dickinson, Rutherford, NJ, USA) and tested by immunofluorescence assay for antibodies to *L*. *pneumophila*, *L*. *anisa*, *L*. *bozemanii*, *L*. *longbeachae*, *L*. *micdadei*, and other AAMs (*15*,*18*), including *Parachlamydia acanthamoeba* (strain BN 9 and "Hall's coccus"), *Afipia birgiae*, *A*. *broomeae*, *A*. *clevelandensis*, *A*. *felis*, *A*. *felis* genospecies A, *Afipia* genospecies 1–3, *A*. *massilliae*, *Azorhizobium caulinodans*, *Bosea eneae*, *B*. *massiliensis*, *B*. *thiooxydans*, *B*. *vestrisii*, *Bradyrhizobium japonicum*, *B*. *liaoningense*, *L*. *quinlivanii*, *L*. *rubrilucens*, *L*. *worsleiensis*, *Mesorhizobium amorphae*, *Rasbo bacterium*, and *Acanthamoeba polyphaga mimivirus* (*13**,**15*). A urine sample was tested for *L*. *pneumophila* serogroup 1 antigen by enzyme-linked immunosorbent assay (*26*) (Binax, Inc., Portland, ME, USA). Data on non-AAMs isolated from BAL or blood cultures were obtained by conventional or specific procedures (culture performed on Löwenstein-Jensen medium, shell-vial culture for cytomegalovirus, and inoculation onto continuous cell lines for indirect immunofluorescence assay for herpes simplex virus). *Mycoplasma pneumonia*, *Chlamydia pneumoniae*, *C*. *psittacci*, *Coxiella burnetii*, influenza viruses A and B, and adenovirus were also identified.

### Main Outcome Measures

Two groups of microorganisms were defined. The first was AAM (*Legionella* spp., *Afipia* spp., *Bosea* spp., *Bradyrhizobium* spp., *Mesorhizobium* spp., *Rasbo bacterium*, *Parachlamydia* spp., and *A*. *polyphaga mimivirus*). The second group was other water-associated microorganisms (*Pseudomonas aeruginosa* and AAM). Diagnosis of AAM infection was classified as having a strong or low level of evidence. The role of an infectious agent in the diagnosis reflected several factors, which included the relationship of the anatomic site of detection to the lung, reliability of the method, and whether the putative agent was a known cause of pneumonia.

Strong evidence for AAM included 1) positive BAL culture, 2) 4-fold increase in antibody titer between acute- and convalescent-phase serum samples or seroconversion from 0 to 1:128 for *L*. *pneumophila*, *L*. *anisa*, *L*. *bozemanii*, *L*. *micdadei*, and *L*. *longbeachae*; and from 0 to 1: 100 for *L*. *quinlivanii*, *L*. *rubrilucens*, *L*. *worsleiensis*, *Afipia* spp., *Bosea* spp., *Bradyrhizobium* spp., *Mesorhizobium* spp., *Parachlamydia* spp., *R*. *bacterium*, and *A*. *polyphaga mimivirus*; and 3) positive results for *L*. *pneumophila* antigen.

A low level of evidence for AAM included a stable antibody titer of >1:256 for *L*. *pneumophila*, *L*. *anisa*, *L*. *bozemanii*, *L*. *micdadei*, and *L*. *longbeachae*; >1:400 for *L*. *quinlivanii*, *L. rubrilucens*, *L. worsleiensis*, *Afipia* spp., *Bosea* spp., *Bradyrhizobium* spp., *Mesorhizobium* spp., *R*. *bacterium*, and *A*. *polyphaga mimivirus*; and >1:200 for *P*. *acanthamoeba*.

### Statistical Analysis

Results are expressed as mean ± standard deviation (SD). Continuous variables were compared by using the Student t-test or the nonparametric Mann Whitney U test when they could not be judged normal. Categoric variables were compared by using the χ^2^ test or Fisher exact test when appropriate. Statistical significance was established at p<0.05. All analyses were performed with SPSS version 10 software (SPSS Inc., Chicago, IL, USA).

## Results

A total of 157 patients with 210 episodes of pneumonia were included in the study. The frequency of pneumonia episodes per patient was 1 in 123 patients, 2 in 22 patients, 3 in 7 patients, 4 in 3 patients, and 5 in 2 patients. The mean ± SD age was 61.6 ± 16.1 years (range 19–99) and 73.8% of the patients were male. Samples were collected in 62 episodes of community-acquired pneumonia, 120 episodes of nosocomial pneumonia, and 28 episodes of mixed pneumonia (community-acquired, complicated with a nosocomial infection). Data collected for 201 episodes of pneumonia indicated a prevalence of 18.4% with chronic obstructive bronchopneumonia, 6.5% with asthma, 0.5% with cystic fibrosis, 41.8% with smoking habits (19% in males vs. 26% in females; p = 0.004), 17.4% with alcohol consumption, 37.3% with immunosuppression (3 cases of prolonged steroid treatment for inflammatory disease [1.5%], 3 HIV infections [1.5%], 2 splenectomies [1.0%], 3 lung transplants [1.5%], 8 kidney transplants [4.0%] and 57 cancers [28.4%]), 6.0% with malnutrition, 5.5% with a history of tuberculosis, 29.8% with previous surgery (62/208), 38.4% with probable or certain inhalation therapy (78/203), and 38.9% with antimicrobial drug therapy for >1 week (70/180). The mean ± SD duration of hospitalization and ventilation was 22.9 ± 32.6 days (range 0–371) and 16.3 ± 19.9 days (range 0–101 days), respectively. Data on antimicrobial treatment before BAL was available for 208 patients. Of these, 116 (55.8%) received an antimicrobial drug, 16 (7.7%) an antiviral drug, and 18 (8.7%) an antimycotic drug.

Some patients had several definite or possible pathogens. A total of 230 documentations corresponding to 40 etiologic agents were identified in 152 (72.4%) of 210 episodes of pneumonia. Eighty-six (41.0%) BAL specimens were contaminated with fungi. Table 1 summarizes the non-AAMs identified as definite (28 in 27 [12.9%] of 210 episodes) or possible (143 in 115 [54.8%] of 210 episodes).

**Table 1:** Identification of 26 nonameba-associated microorganisms in 210 episodes of pneumonia

Microorganism		Definite,* no. (%)	Possible,† no. (%)	Total, no. (%)
Community-acquired pneumonia				
Bacteria				
	*Acinetobacter baumanii*		2 (1.4)	2 (1.2)
	*Chlamydia pneumoniae*	1 (3.6)	2 (1.4)	3 (1.8)
	*C*. *psittacci*	1 (3.6)	2 (1.4)	3 (1.8)
	*Enterobacter cloacae*		1 (0.7)	1 (0.6)
	*E*. *faecalis*		1 (0.7)	1 (0.6)
	*Escherichia coli*	2 (7.1)	2 (1.4)	4 (2.3)
	*Haemophilus influenzae*	1 (3.6)	3 (2.1)	4 (2.3)
	*Mycobacterium tuberculosis*	1 (3.6)		1 (0.6)
	*Pseudomonas aeruginosa*	1 (3.6)	2 (1.4)	3 (1.8)
	*Serratia marcescens*		1 (0.7)	1 (0.6)
	*Staphylococcus aureus*	1 (3.6)	5 (3.5)	6 (3.6)
	*Streptococcus agalactiae*		1 (0.7)	1 (0.6)
	*S*. *pneumoniae*		3 (2.1)	3 (1.8)
Fungi				
	*Pneumocystis carinii*	3 (10.7)		3 (1.8)
	Viruses‡			
	Cytomegalovirus		2 (1.4)	2 (1.2)
	Herpes simplex virus 1		4 (2.8)	4 (2.3)
Ventilator-associated pneumonia				
Bacteria				
	*A*. *baumanii*		1 (0.7)	1 (0.6)
	*Balneatrix alpica*	1 (3.6)		1 (0.6)
	*C*. *pneumoniae*		1(0.7)	1 (0.6)
	*Citrobacter koseri*		1 (0.7)	1 (0.6)
	*Clostridium freundii*		1 (0.7)	1 (0.6)
	*Coxiella burnetii*		1 (0.7)	1 (0.6)
	*Enterobacter aerogenes*		4 (2.8)	4 (2.3)
	*E*. *cloacae*		5 (3.5)	5 (2.9)
	*E*. *coli*	2 (7.1)	6 (4.2)	8 (4.7)
	*H*. *influenzae*		1 (0.7)	1 (0.6)
	*Proteus mirabilis*		3 (2.1)	3 (1.8)
	*Pseudomonas aeruginosa*	9 (32.1)	31 (21.7)	40 (23.4)
	*Raoultella ornithinolytica*		1 (0.7)	1 (0.6)
	*S*. *marcescens*		2 (1.4)	2 (1.2)
	*S*. *aureus*	2 (7.1)	21 (14.7)	23 (13.6)
	*S*. *epidermidis*	1 (3.6)	1 (0.7)	2 (1.2)
	*Stenotrophomonas maltophilia*		5 (3.5)	5 (2.9)
	*S*. *agalactiae*		1 (0.7)	1 (0.6)
	*S*. *pneumoniae*	1 (3.6)	2 (1.4)	3 (1.8)
Fungi				
	*Candida albicans*	1 (3.6)		1 (0.6)
Viruses‡				
	Cytomegalovirus		11 (7.7)	11 (6.4)
	Herpes simplex virus 1		13 (9.1)	13 (7.6)
Total		28 (100.0)	143 (100.0)	171 (100.0)

*Detection of *M*. *tuberculosis* or *P*. *carinii* by bronchioalveolar lavage (BAL); simultaneous positive culture with BAL and blood culture; positive for influenza viruses A and B, adenovirus, or *C*. *burnetii* (immunoglobulin G2 [IgG2] titer >1:200, IgM2 >1:50); 4-fold increase in antibody titer between acute- and convalescent-phase serum; or seroconversion from 0 to 1:128 for *C*. *psittacci*, from 0 to 1:256 for *C*. *pneumoniae*, from 0 to 1 for *M*. *pneumoniae*, and from 0 to 1:100 for *B*. *alpica*. †Detection of a potentially pathogenic microorganism (*M*. *tuberculosis* and *P*. *carinii*) by BAL and single or stable antibody titer >1:512 for *Chlamydia* spp., >1:2 for *M*. *pneumoniae*, and >1:400 for *B*. *alpica*. ‡No epidemic of influenza virus A/B or adenovirus was observed during the 18-month study period.

Laboratory investigations for AAMs detected 59 diagnoses in 40 (19.0%) patients. More than 1 AAM was observed in 56 episodes of pneumonia (26.7%); 39 (18.6%) had 2 AAMs, 11 (5.2%) had 3 AAMs, 3 (1.4%) had 4 AAMs, 2 (1.0%) had 5 AAMs, and 1 (0.5%) had 6 AAMs. Ten patients had serologic evidence of mixed infections with AAMs. Of the 40 patients with documented AAM infections, 18 (8.6% of our series) had evidence of AAMs (Table 2); 9 of these patients showed a high level of evidence. Evidence of pneumonia caused by unique AAMs was obtained in 13 patients. Of these, 5 had pneumonia caused by *A*. *polyphaga mimivirus*, 5 caused by *L*. *pneumophila*, 2 caused by *L*. *bozemanii*, and 1 caused by *Parachlamydia* sp. Mixed infections in these patients with 2, 3, and 5 AAMs were observed in 1, 2, and 2 patients, respectively. A unique AAM was observed in 13 patients (5 with *A*. *polyphaga mimivirus*, 5 with *L*. *pneumophila*, 2 with *L*. *bozemanii*, and 1 with *Parachlamydia* sp.).

**Table 2:** Description of 18 cases of pneumonia with only identification of ameba-associated microorganisms

High level of evidence	Low level of evidence	No.
*Acanthamoeba polyphaga mimivirus*		5*
*Legionella pneumophila*		1†
*L*. *pneumophila*	*L*. *anisa*	1
*Parachlamydia* sp.	*Bosea thiooxydans*, *L*. *boozemanii*	1
*Bosea massiliensis*, *L*. *quinlivanii*, *L*. *rubrilucens*, *L*. *worsleiensis*	*Bradyrhizobium japonicum*	1
	*L*. *pneumophila*	4*
	*L*. *bozemanii*	2
	*Parachlamydia* sp.	1
	*B*. *thiooxydans*, *B*. *japonicum*, *Rasbo bacterium*	1
	*L*. *pneumophila*, *L*. *rubrilucens*, *B*. *massiliensis*, *B*. *japonicum*, *R*. *bacterium*	1

*One case of community-acquired pneumonia. †Community-acquired pneumonia.

A total of 22 (10.5%) of 210 episodes of pneumonia were observed in which both AAMs and conventional microorganisms were detected (Table 3). Six patients had diagnoses of AAM infection with a high level of evidence. Three of these 6 patients also had definite diagnoses of pneumonia caused by AAMs, and 3 others had a possible diagnosis of pneumonia caused by non-AAMs. Sixteen patients had diagnoses of pneumonia caused by AAMs with a low level of evidence. Three of these patients also had definite diagnoses of pneumonia caused by non-AAMs, and 13 had possible diagnoses of pneumonia caused by non-AAMs.

**Table 3:** Description of 22 cases of pneumonia with identification of ameba-associated and nonameba-associated microorganisms*

Ameba-associated level of evidence		Nonameba-associated identification	
High	Low	Definite	Possible
*Acanthamoeba polyphaga mimivirus†*		*Staphylococcus aureus†*	
*A*. *polyphaga mimivirus*		*Chlamydia pneumoniae*	
*Mesorhizobium amorphae*, *Rasbo bacterium*		*Pseudomonas aeruginosa*, *Balneatrix alpica*	
*Legionella pneumophila*			HSV1
*L*. *anisa*†			*Serratia marcescens†*
*A*. *polyphaga mimivirus*			*P*. *aeruginosa*
	*L*. *pneumophila*†	*P*. *aeruginosa*†	
	*L*. *pneumophila*	*Candida albicans*	
	*Bosea massiliensis*, *Bradyrhizobium japonicum*†	*Escherichia coli†*	
	*Parachlamydia* sp.		*Streptococcus pneumoniae*
	*A*. *polyphaga mimivirus*		*Enterobacter cloacae*, *C*. *pneumoniae*
	*A*. *polyphaga mimivirus*		CMV
	*A*. *polyphaga mimivirus*		*E*. *cloacae*
	*A*. *polyphaga mimivirus*		*E*. *aerogenes*, *P*. *aeruginosa*
	*A*. *polyphaga mimivirus*		*P*. *aeruginosa*
	*A*. *polyphaga mimivirus*		*S*. *aureus*
	*Bosea thiooxydans*, *A*. *polyphaga mimivirus*		*S*. *aureus*
	*B*. *massiliensis*, *B*. *japonicum*, *R*. *bacterium*		*Proteus mirabilis*, *S*. *aureus*
	*B*. *massilensis*, *B*. *japonicum*		HSV1
	*Bradyrhizobium liaoningense*		*S*. *aureus*
	*B*. *liaoningense*		*P*. *aeruginosa*
	*B*. *liaoningense*		*Stenotrophomonas maltophilia*

*HSV1, herpes simplex virus 1; CMV, cytomegalovirus. †Community-acquired pneumonia.

Fifteen patients were identified as having definite cases of pneumonia caused by AAMs. This subgroup (of whom 1 had a definite diagnosis of *S*. *aureus* infection and 1 of *C*. *pneumoniae* infection), included 8 patients with pneumonia caused by *A*. *polyphaga mimivirus*, 3 with pneumonia caused by *L*. *pneumophila*, and 5 who seroconverted. Those who seroconverted included any patient with seroconversion for *L*. *anisa*, *Parachlamydia* sp., *B*. *massiliensis*, *L*. *worsleiensis*, *L*. *quinlivanii*, *L*. *rubrilucens*, *M*. *amorphae*, and *R*. *bacterium*. In addition, 1 who seroconverted also had a diagnosis of infection with *P*. *aeruginosa* and *B*. *alpica*. Eleven patients had possible infections with *Legionella* sp. (*L*. *pneumophila* in 7, *L*. *bozemanii* in 3, and *L*. *anisa* in 1), and 19 patients had possible infections with atypical organisms (*A*. *polyphaga mimivirus* in 7, *B*. *japonicum* in 6, *B*. *massiliensis* in 4, *B*. *liaoningense* in 3. *B*. *thiooxydans* in 3, *R*. *bacterium* in 3, *Parachlamydiae* sp. in 2, and *L*. *rubrilucens* in 1).

The frequency of infections with AAMs is summarized in Table 4. *A*. *polyphaga mimivirus*, which was identified in 15 (7.1%) of 210 episodes of pneumonia, was the most common AAM. *Legionella* sp. were identified in 14 episodes. Three of these patients had mixed infections (*L*. *pneumophila* and *L*. *anisa* in 1, *L*. *pneumophila* and *L*. *rubrilucens* in 1, and *L*. *quilivanii*, *L*. *rubrilucens*, and *L*. *worsleiensis* in 1). *L*. *pneumophila*, which was identified in 10 (4.8%) of 210 episodes, was the second most frequently documented AAM. *Bradyrhizobium* sp. was identified in 9 patients; 6 of them were also infected with *B*. *japonicum*. Five of 8 patients infected with *Bosea* sp. were also infected with *B*. *massiliensis*. Four patients had serologic evidence of mixed infection with *B*. *japonicum* and *B*. *massiliensis*. The 7 most common etiologic agents were *P*. *aeruginosa* (20.5%), *S*. *aureus* (13.8%), herpes simplex virus (8.1%), *A*. *polyphaga mimivirus* (7.1%), cytomegalovirus (6.2%), *Escherichia coli* (5.7%), and *L*. *pneumophila* (4.8%). If one considers only diagnoses with a high level of evidence, the 4 most common etiologic agents were *P*. *aeruginosa* (4.8%), *A*. *polyphaga mimivirus* (3.8%), *E*. *coli* (1.9%), and *L*. *pneumophila* (1.4%).

**Table 4:** Identification of ameba-associated microorganisms in pneumonia and level of evidence

Microorganism	High, no. (%)	Low, no. (%)	Total, no. (%)
Community-acquired pneumonia			
Bacteria			
	*Bosea massiliensis*		1 (2.5)	1 (1.7)
	*Bradyrhizobium japonicum*		1 (2.5)	1 (1.7)
	*Legionella anisa*	1 (5.3)		1 (1.7)
	*L*. *pneumophila*	1 (5.3)	2 (5.0)	3 (5.1)
Virus				
	*Acanthamoeba polyphaga mimivirus*	2 (10.5)		2 (3.4)
Ventilator-associated pneumonia			
Bacteria			
	*B*. *massiliensis*	1 (5.3)	3 (7.5)	4 (6.8)
	*B*. *thiooxydans*		3 (7.5)	3 (5.1)
	*B*. *japonicum*		5 (12.5)	5 (8.5)
	*B*. *liaoningense*		3 (7.5)	3 (5.1)
	*L*. *anisa*		1 (2.5)	1 (1.7)
	*L*. *bozemanii*		3 (7.5)	3 (5.1)
	*L*. *pneumophila*	2 (10.5)	5 (12.5)	7 (11.9)
	*L*. *quinlivanii*	1 (5.3)		1 (1.7)
	*L*. *rubrilucens*	1 (5.3)	1 (2.5)	2 (3.4)
	*L*. *worsleiensis*	1 (5.3)		1 (1.7)
	*Mesorhizobium amorphae*	1 (5.3)		1 (1.7)
	*Parachlamydiae acanthamoebae*	1 (5.3)	2 (5.0)	3 (5.1)
	*Rasbo bacterium*	1 (5.3)	3 (7.5)	4 (6.8)
Virus			
	*A*. *polyphaga mimivirus*	6 (31.6)	7 (17.5)	13 (22.0)
Total	19 (100)	40 (100)	59 (100)

A diagnosis was more frequent in a nosocomial context than outside a hospital (79.1% vs. 54.8%, p<10^–3^), especially for *P*. *aeruginosa* (p<10^–6^). Water-associated microorganisms were less likely to be identified in a community-acquired context than in a nosocomial context (30% vs. 50%, p = 0.005). Duration of hospitalization and ventilation were longer for patients infected with the water-associated microorganisms than for patients not infected (29 days vs. 19 days p = 0.015 and 21 days vs. 13 days, p = 0.008, respectively). Therapy with antimicrobial agents and a history of cancer were also more frequent in patients infected with water-associated microorganisms (54% vs. 30%, p = 0.001 and 39% vs. 22%, p = 0.014, respectively). Patients who seroconverted for *A*. *polyphaga mimivirus* used alcohol more frequently than others in the study (44% vs. 18%, p = 0.05).

## Discussion

We conducted this study to determine the role of AAMs as causative agents of pneumonia in patients in an ICU. Concerns have been reported about the role of inline medication nebulizers contaminated with water-associated microorganisms, AAMs, or both (*11**,**14**,**15*). Other microorganisms, including *Legionella*-like amebal pathogens, *P*. *acanthamoeba*, *Afipia* sp., *Bosea* sp., *Bradyrhizobium* sp., *Mesorhizobium* sp., and *A*. *polyphaga mimivirus*, have also been reported (*14**,**19**,**20**,**27*). Our results indicate that AAMs represented 25.3% (59/233) of all documented causes of pneumonia and that 19.0% (40/210) of all episodes of pneumonia were associated with AAMs.

Marrie et al. reported that *Legionella*-like amebal pathogens might play a role in pneumonia, usually as co-infecting organisms (*18*). In 18 patients (8.6%), the role of AAMs were well documented. Nine of these patients had a high level of evidence for AAMs. Both conventional microorganisms and AAMs were implicated in 22 (10.5%) cases. However, 6 of them had high levels of evidence for AAM infections. Three of these 6 patients had documented infections with *L*. *pneumophila*, *L*. *anisa*, and *A*. *polyphaga mimivirus* and low levels of infection with herpes simplex virus, *S*. *marcescens*, and *P*. *aeruginosa*. The serologic evidence obtained from these patients demonstrates only that they were infected by these bacteria or a cross-reactive microorganism, not that these bacteria caused their pneumonia. However, the fact that only 8.6% had only indirect evidence of AAM infection raises questions about the potential pathogenic role of AAMs in pneumonia.

*A*. *polyphaga mimivirus* was the fourth most common cause of pneumonia in our study. This finding suggests that this organism may be clinically relevant. However, several lines of evidence now indicate that ameba-resisting microorganisms other than *Legionella* sp. are associated with both community- and hospital-acquired pneumonia (*19**,**28*). La Scola et al. (*13*) and Marrie et al. (*18*) have reported that the seroprevalence of *Legionella* was higher than that of other AAMs. Except for *L*. *pneumophila* findings, our results agree. The seroprevalence of *Legionella* (7.1%) in our series was lower than that reported by others in community-acquired (9.7%) and hospital-acquired (19.2%) pneumonia (*13*). However, this prevalence was significantly higher (p<0.002) than that observed (2.3%) in a healthy control population (*13*). These data also suggest that some patients with ventilator-associated pneumonia might have been in contact with *A*. *polyphaga mimivirus* or other cross-reactive antigens. These results raise questions about the pathogenic potential of the largest virus known or cross-reactive antibodies to an unknown organism (*13*).

We observed a significantly lower prevalence of seroconversion (p<10^-2^) for other AAMs than was found in a previous series: 32 (15.2%) of 210 serologically diagnosed cases of AAM pneumonia compared with 12 (40.0%) patients hospitalized in another ICU (*15*). The serologic evidence (e.g., seroconversion) obtained in this study strongly suggests that this patient population may have been exposed to the most common water ameba-associated bacteria in their environment (*15*). No environmental investigations were performed in our epidemiologic survey. The lower seroprevalence of AAMs in our patients suggest that they may have had less exposure in our hospital ICU compared with that observed in previous studies.

Interest in free-living amebas has grown over the last decade with reports of their pathogenic potential (*11**,**29*) and the role of amebas as reservoirs for *L*. *pneumophila* and other AAMs (*12**–**15**,**17**,**27**,**30**,**31*). Since respiratory care protocols use only sterile water, 2 possible routes of infection with AAMs include a breach in protocol enforcement and handborne AAMs. Adherence to these protocols and use of water filters ensures better protection of water supplies, as is the case in our ICU.

An interesting finding was that ≈44.8% of the patients with severe pneumonia had mixed causes. AAM was implicated in 12.9% of these patients. Fagon et al. reported that only one third of the therapeutic regimens proposed for pneumonia patients needing ventilators were effective (*32*). Because the recommended empiric approaches in guidelines are based on microbial patterns derived from several epidemiologic surveys (*33*), clinicians need to know the local, regional, and global patterns of microbial populations and the possibility of emerging pathogens such as AAMs. If these microorganisms are human pathogens, they will influence the choice of antimicrobial drugs for empiric treatment because most are resistant to carboxypenicillins, ureidopenicillins, third-generation cephalosporins, and fluoroquinolones, which are commonly used in the ICU.

AAMs may cause ventilator-associated pneumonia and should be suspected when results of conventional microbiologic investigations are negative (*11**,**15**,**19**,**28*). A diagnosis is rarely available at the time treatment with antimicrobial agents is begun. Thus, the prevailing situation warrants better diagnosis of pneumonia and identification of new lung pathogens such as AAMs. Recognizing the emerging pathogens responsible for pneumonia should be a major public health concern because the knowledge of predominant microbial patterns will help provide the basis for rational empiric antimicrobial treatment.

## References

[1] Bartlett JG, Dowell SF, Mandell LA, File TM Jr, Musher DM, Fine MJ. Practice guidelines for the management of community-acquired pneumonia in adults. Infectious Diseases Society of America. Clin Infect Dis. 2000;31:347–82. 10.1086/313954 10987697 PMC7109923

[2] Marrie TJ. Community-acquired pneumonia in the elderly. Clin Infect Dis. 2000;31:1066–78. 10.1086/318124 11049791

[3] Ruiz M, Ewing S, Torres A, Arancibia F, Francesc M, Mensa J, Severe community-acquired pneumonia. Risk factors and follow-up epidemiology. Am J Respir Crit Care Med. 1999;160:923–9. 10471620 10.1164/ajrccm.160.3.9901107

[4] Cook D. Ventilator associated pneumonia: perspectives on the burden of illness. Intensive Care Med. 2000;26:31–7. 10.1007/s001340051116 10786956

[5] Marrie TJ, Durant H, Yates L. Community-acquired pneumonia requiring hospitalization: 5 years prospective study. Rev Infect Dis. 1989;11:586–99. 10.1093/clinids/11.4.586 2772465

[6] Papazian L, Fraisse A, Garbe L, Zandotti C, Thomas P, Saux P, Cytomegalovirus: an unexpected cause of ventilator-associated pneumonia. Anesthesiology. 1996;84:280–7. 10.1097/00000542-199602000-00005 8602657

[7] Chastre J, Fagon JY. Ventilator-associated pneumonia. Am J Respir Crit Care Med. 2002;165:867–903. 11934711 10.1164/ajrccm.165.7.2105078

[8] Bruynseels P, Jorens PG, Demey HE, Goossens H, Pattyn SR, Elseviers MM, Herpes simplex virus in the respiratory tract of critical care patients: a prospective study. Lancet. 2003;362:1536–41. 10.1016/S0140-6736(03)14740-X 14615108

[9] Anaissie EJ, Penzak SR, Dignani MC. The hospital water supply as a source of nosocomial infections: a plea for action. Arch Intern Med. 2002;162:1483–92. 10.1001/archinte.162.13.1483 12090885

[10] Rutala WA, Weber DJ. Water as a reservoir of nosocomial pathogens. Infect Control Hosp Epidemiol. 1997;18:609–16. 10.1086/647684 9309431

[11] Greub G, Raoult D. Microorganisms resistant to free-living amoebae. Clin Microbiol Rev. 2004;17:413–33. 10.1128/CMR.17.2.413-433.2004 15084508 PMC387402

[12] La Scola B, Audic S, Robert C, Jungang L, de Lamballerie X, Drancourt M, A giant virus in amoebae. Science. 2003;299:2033. 10.1126/science.1081867 12663918

[13] La Scola B, Marrie TJ, Auffray JP, Raoult D. Mimivirus in pneumonia patients. Emerg Infect Dis. 2005;11:449–52. 15757563 10.3201/eid1103.040538 PMC3298252

[14] La Scola B, Mezi L, Auffray JP, Berland Y, Raoult D. Patients in the intensive care unit are exposed to amoeba-associated pathogens. Infect Control Hosp Epidemiol. 2002;23:462–5. 10.1086/502086 12186213

[15] La Scola B, Boyadjiev I, Greub G, Khamis A, Martin C, Raoult D. Amoeba-resisting bacteria and ventilator-associated pneumonia. Emerg Infect Dis. 2003;9:815–21. 12890321 10.3201/eid0907.030065 PMC3023432

[16] Raoult D, Audic S, Robert C, Abergel C, Renesto P, Ogata H, The 1.2-megabase genome sequence of Mimivirus. Science. 2004;306:1344–50. 10.1126/science.1101485 15486256

[17] La Scola B, Mezi L, Weiller PJ, Raoult D. Isolation of *Legionella anisa* using an amoebic coculture procedure. J Clin Microbiol. 2001;39:365–6. 10.1128/JCM.39.1.365-366.2001 11136802 PMC87733

[18] Marrie TJ, Raoult D, La Scola B, Birtles RJ, de Carolis E; The Canadian Community-Acquired Pneumonia Study Group. *Legionella*-like and other amoebal pathogens as agents of community-acquired pneumonia. Emerg Infect Dis. 2001;7:1026–9. 10.3201/eid0706.010619 11747734 PMC2631911

[19] Greub G, Berger P, Papazian L, Raoult D. *Parachlamydiaceae* as rare agents of pneumonia. Emerg Infect Dis. 2003;9:755–6. 12781026 10.3201/eid0906.020613 PMC3000139

[20] Greub G, Raoult D. *Parachlamydiaceae*: potential emerging pathogens. Emerg Infect Dis. 2002;8:625–30. 12023921 10.3201/eid0806.010210 PMC2738484

[21] Meduri GU, Mauldin GL, Wunderink RG, Leeper KV, Jones CB, Tolley E, Causes of fever and pulmonary densities in patients with clinical manifestations of ventilator-associated pneumonia. Chest. 1994;106:221–35. 10.1378/chest.106.1.221 8020275

[22] Bernard GR, Artigas A, Brigham KL. the Consensus Committee. The American-European consensus conference on ARDS: definitions, mechanisms, relevant outcomes, and the clinical trial co-ordination. Am J Respir Crit Care Med. 1994;149:818–24. 7509706 10.1164/ajrccm.149.3.7509706

[23] Winn WC. Legionella. In: Press A, Murray PR, Baron EJ, Pfaller MA, Tenover FC, Yolken RH, editors. Manual of clinical microbiology. 6th ed. Washington: American Society for Microbiology; 1995. p. 533–44.

[24] La Scola B, Michel G, Raoult D. Isolation of *Legionella pneumophila* by centrifugation of shell vial cell cultures from multiple liver and lung abscesses. J Clin Microbiol. 1999;37:785–7. 9986854 10.1128/jcm.37.3.785-787.1999 PMC84555

[25] Rowbotham TJ. Isolation of *Legionella pneumophila* from clinical specimens via amoeba and the interaction of those and other isolates with amoebae. J Clin Pathol. 1983;36:978–86. 10.1136/jcp.36.9.978 6350372 PMC498455

[26] Berdal BP, Farshy CE, Feeley JC. Detection of *Legionella pneumophila* antigen in urine by enzyme-linked-immunospecific assay. J Clin Microbiol. 1979;9:575–8. 383744 10.1128/jcm.9.5.575-578.1979 PMC275350

[27] La Scola B, Raoult D. *Afipia felis* in hospital water supply in association with free-living amoebae. Lancet. 1999;353:1330. 10.1016/S0140-6736(99)00906-X 10218538

[28] Martin WJ, Smith TF. Rapid detection of cytomegalovirus in brochoalveolar lavage specimens by a monoclonal antibody method. J Clin Microbiol. 1986;23:1006–8. 3011853 10.1128/jcm.23.6.1006-1008.1986 PMC268781

[29] Szénasi Z, Yagiat EK, Nagy E. Isolation, identification and increasing importance of "free-living" amoebae causing human disease. J Med Microbiol. 1998;47:5–16. 10.1099/00222615-47-1-5 9449945

[30] Winiecka-Krusnell J, Linder E. Free-living amoebae protecting *Legionella* in water: the tip of an iceberg? Scand J Infect Dis. 1999;31:383–5. 10.1080/00365549950163833 10528878

[31] Winiecka-Krusnell J, Linder E. Bacterial infections of free-living amoebae. Res Microbiol. 2001;152:613–9. 10.1016/S0923-2508(01)01240-2 11605981

[32] Fagon JY, Chastre J, Hance AJ, Domart Y, Trouillet JL, Gilbert C. Evaluation of clinical judgment in the identification and treatment of nosocomial pneumonia in ventilated patients. Chest. 1993;103:547–53. 10.1378/chest.103.2.547 8432152

[33] British Thoracic Society Standards of Care Committee. BTS guidelines for the management of community acquired pneumonia in adults. Thorax. 2001;56(Suppl 4):1–64. 10.1136/thorax.56.suppl_1.i1 11713364 PMC1765992

